# Evaluation of Parameters and Nozzle Tip Damage after Clinical Use of Three Hydrophilic Intraocular Lens Injector Models

**DOI:** 10.1155/2024/2360368

**Published:** 2024-05-30

**Authors:** Lu Zhang, Sonja Schickhardt, Patrick Merz, Gerd Uwe Auffarth

**Affiliations:** David J Apple Center for Vision Research, Department of Ophthalmology, University Hospital Heidelberg, Im Neuenheimer Feld 400, Heidelberg 69120, Germany

## Abstract

**Purpose:**

To assess the nozzle tip damage and the parameters of three different hydrophilic intraocular lens (IOL) injector models.

**Methods:**

After routine cataract surgeries at the University Eye Hospital Heidelberg, all the used IOL injectors were collected from the operating room and sent to our laboratory. Nozzle tip damage was assessed under a microscope and graded as follows: no damage (grade 0), slight scratches (1), deep scratches (2), extensions (3), cracks (4), and bursts (5). Each damage grade was assigned a score from 0 to 5, and the total damage score for each injector system was calculated and compared. Nozzle tip parameters (diameters and areas), plunger tip parameters, and tip angles were also measured in each model.

**Results:**

The damage scores were (median, Q3-Q1): 1 (1-1) for Accuject, 1 (1-1) for Bluemixs, and 1 (1-1) for RayOne. There was no statistically significant difference in the damage scores between the study groups (*P* > 0.05). The outer cross-sectional vertical and horizontal diameters were 1.69 and 1.69 mm for Accuject, 1.69 and 1.69 mm for Bluemixs, and 1.70 and 1.71 mm for RayOne. Plunger tip areas were 0.78 mm^2^ for Accjuect, 0.74 mm^2^ for Bluemixs, and 0.43 mm^2^ for RayOne. Plunger tip area/inner cross-sectional area of the nozzle tip (%) was 31.2% for RayOne, 66.7% for Accuject, and 63.8% for Bluemixs. The tip angles for three injector models were 56° (Accuject), 56° (Bluemixs), and 44° (RayOne).

**Conclusions:**

All the injector models showed mild to moderate damage to the nozzle tip after IOL implantation, even with smaller diameter tips. RayOne resulted in the lowest ratio between plunger tip area and inner cross-sectional area of the nozzle tip and a better distribution of damage categories than the other two groups. All three injector models had relatively small tip parameters. If smaller incisions are required in certain patients, smaller tip parameters should be considered.

## 1. Introduction

Since Ridley implanted the first intraocular lens (IOL), cataract surgery has evolved significantly [1]. The IOL is inserted into the eye to replace the natural lens in most circumstances. Acrylic IOLs are currently the dominant IOLs worldwide [2] and can be further divided into hydrophobic acrylic IOLs and hydrophilic acrylic IOLs. Hydrophilic acrylic IOLs are widely used in Europe because they are easy to handle and can be inserted through incisions of less than 2 mm [3].

The implantation of IOLs requires IOL injector systems. Injector systems usually consist of three parts: the injector body, the plunger, and the cartridge. The injector body could be made of metal or plastic. Although the plastic injector body tends to be more popular in the market, the reusable injector body (made of metal) is still in use today [4]. The cartridge, made of plastic, was disposable and contained a “lens loading part” for IOL loading and a “nozzle tip” for insertion.

The shape of the nozzle tip is usually round, oval, or hexagonal. However, several studies [5, 6] have associated hexagonal nozzle tips with an increased risk of plunger override and the formation of linear deposits on the surface of the IOL optic. As a result, the hexagonal nozzle tip is being replaced by its round and oval counterparts.

A few studies [6–8] have correlated IOL surface abnormalities after IOL implantation with injector cartridge damage. In an earlier study, a correlation between IOL surface abnormalities and defects on the inner walls of the injector cartridge has been reported [7]. Another study also pointed out that [8], the inner part of the cartridge is likely the source of the deposits on the IOL surface. Some of these surface abnormalities may persist for more than a year and even lead to further complications [8]. Furthermore, it has been suggested in the literature that hydrophilic IOLs are more prone to IOL damage than other IOL types [6].

Although IOL delivery with the injector system has been successful in most cases, inadvertent events still occur during the procedure. Adverse events during implantation that are related to the damage of the nozzle tips may include haptic-optic adhesion, trapped posterior haptics, IOL attachment to the plunger, and even the plastic part of the injector has been observed in the anterior chamber [9, 10].

Incision size has always been a driving force for injector innovation. Nozzle tip materials, designs, geometric shapes, internal and external dimensions, and implantation method (push mode or screw mode) all contribute to incision size [10, 11].

Therefore, a thorough understanding of injector tips and the examination of injector tip damage after IOL implantation may provide insight into reducing incision sizes, eliminating IOL surface abnormalities, and promoting a smoother IOL implantation procedure.

Hydrophobic and hydrophilic IOLs are currently the most commonly used IOL models worldwide. It would be beneficial to gain a better understanding of the IOL injector models of these two IOL categories. In our previous study [12], we evaluated nozzle tip damage in a series of hydrophobic IOL injectors using our self-developed damage scale, the Heidelberg Score for IOL Injector Damage (HeiScore). However, to the best of our knowledge, no evaluation of nozzle tip damage of hydrophilic IOL injectors has been performed to date. Therefore, in this study, we evaluated hydrophilic IOL injectors of three injector models using our self-developed damage scale—the Heidelberg Score for IOL Injector Damage (HeiScore).

## 2. Materials and Methods

### 2.1. Collection of IOL Injectors

**Figure 1:**
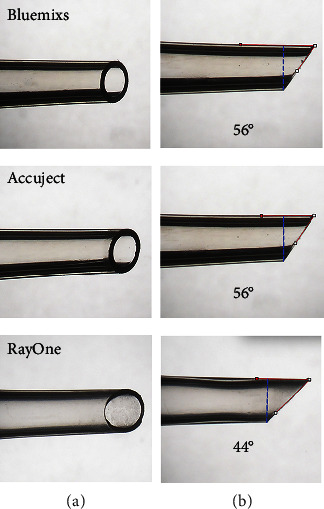
Representative microscopic images of nozzle tips of three unused IOL injector models. (a) Nozzles of each injector model in axial view. (b) Nozzles in profile view. The angle of each nozzle is indicated by the red line, the position of the cross-section surface is indicated by the blue dashed line.

The same approach was used to obtain the IOL injectors as in our earlier research [12]. In a series of routine, uncomplicated cataract surgeries at the University Eye Hospital Heidelberg, 77 IOL injectors from three different injector models—16 from Accuject, 46 from Bluemixs, and 15 from RayOne—were used for IOL implantation. Supplementary Table 1 provides an overview of the IOL articles used in this research. One experienced surgeon (GUA) performed all the surgical operations. The microscopic view of a single, unused IOL injector from each model is shown in Figure 1. 2.4 or 2.5 mm clear corneal incisions were created in each cataract case. The power of the IOLs in this study varied from +15D to +26D. All the hydrophilic IOL models were immersed in liquids in their original packaging prior to implantation. To prime the injectors, ophthalmic viscosurgical device (OVD) of 1% sodium hyaluronate (ProVisc, Alcon Laboratories Inc., Fort Worth, Texas, USA) was used in all cases. A gross examination was carried out under the operating microscope after each implantation to determine any IOL damage.

The used injectors were taken from the operating room after each operation and sent to our laboratory. The nozzles were rinsed in distilled water for ten minutes, then air dried to remove any remaining OVD. When handling, care was made to avoid scratching the injector nozzle tips.

### 2.2. Assessment of Nozzle Tip Parameters

To obtain a cross-sectional surface for each injector system, the nozzle tip of each injector model was cut with a razor at the starting point of the bevel angle. The cross-sectional surfaces were photographed under the microscope (Olympus BX50, Olympus K. K.). The plunger tip of each injector model was also cut out and photographed under the microscope. ImageJ (1.52a, National Institutes of Health in Bethesda, Maryland, USA) was used to determine the cross-sectional areas' diameters. The measurements were calibrated using a picture of an IOL captured at the same magnification as the cross-sectional surfaces. First, the diameter of the IOL optic (6 mm) on the image was measured in pixels as a reference. Second, the mathematical conversion between pixels and millimeters was determined. Third, using the arithmetic ratio, the diameter of the cross-sectional surfaces on the image was first measured in pixels and then converted to millimeters. The inner and outer cross-sectional areas of the nozzle tips were calculated using the formula: *A* = *πab* (*a* = horizontal cross-sectional radius, *b* = vertical cross-sectional radius). The diameters of the plunger tips were also measured using the same arithmetic ratio. The areas of the plunger tips were then measured using ImageJ. The tip angles of three injector models were also measured using ImageJ (Figure 1).

### 2.3. Heidelberg Score for IOL Injector Damage (HeiScore)

After air drying, the nozzles were examined under a microscope (Olympus BX50, Olympus K.K.). Each nozzle tip was first inspected in the “bevel down” and “bevel up” orientations, followed by the two lateral orientations to identify any damage to the nozzle tip. The damage was then photographed under the microscope. Based on our scoring system [12], the damage observed on the injectors was classified into the following six grades. Supplementary Figure 1 [12] shows example images for each classification of nozzle tip damage. It should be noted that when more than one damage category is observed at the nozzle tip, the damage assessment will be based on the category with the greatest extent of damage. For example, if both grade 3 and grade 4 damage are observed, only grade 4 is included in the calculation:

  Grade 0: There is no damage observed on the nozzle tips.
  Grade 1: There is slight scratch—fine stress lines on the inner tube or/and slight discontinuity at the nozzle tips.
  Grade 2: There is deep scratch—deep stress lines on the inner tube or/and obvious discontinuity at the nozzle tips.
  Grade 3: There is extension of “deep stress line,” but the deep stress line does not reach the level of full thickness tube crack.
  Grade 4: There is crack—full thickness crack of the injector tubes.
  Grade 5: There is burst of the injector tubes.

Each damage grade has been assigned a score from 0 to 5. For example, grade 0 is assigned a score of 0 and grade 5 is assigned a score of 5. The total damage score for each injector system was the sum of the scores for all injectors in that model. The total damage scores for each injector system were then compared.

### 2.4. Statistical Analyses

To ascertain whether the damage scores and diopters of the IOLs in each injector group were normally distributed, the Shapiro–Wilk test was used. To check for significant differences in damage scores between various injector models, the Kruskal–Wallis H test with Dunn's post hoc comparison was used. The Kruskal–Wallis H test also was used to determine whether there were any significant differences in the diopters of the IOLs between the groups. All statistical tests were conducted using GraphPad Prism (version 9.0, GraphPad Software, SD, USA), and a *P* value of less than 0.05 was considered statistically significant.

## 3. Results

### 3.1. Damage Scores in Each Group

**Figure 2:**
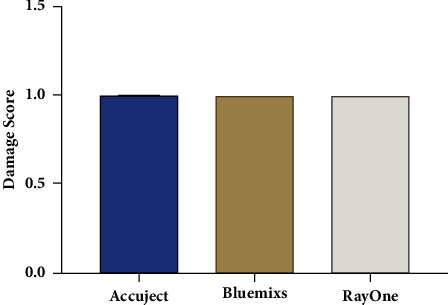
The damage assessment results for each IOL injector model (median with interquartile range).

Results of damage score for each IOL injector model are summarized in Figure 2. Data were expressed as median with interquartile range. Results of damage score model were (median, Q3-Q1): 1 (1-1) for Accuject, 1 (1-1) for RayOne and 1 (1-1) for Bluemixs. All three groups produced comparable results in terms of damage score. No statistically significant difference was observed between any of the study groups (*P* > 0.05). No statistically significant difference was observed across three groups in terms of the dioptric powers of the IOLs (*P* > 0.05, see Supplementary Figure 2).

#### 3.1.1. Distribution of Damage Profiles

**Figure 3:**
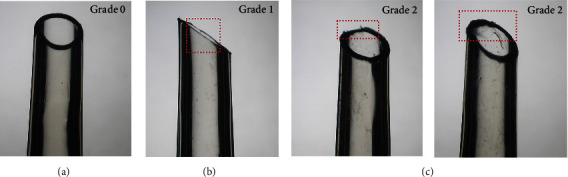
Representative microscopic images of each damage classification in IOL injector models. (a) Showed images of “no damage.” (b). Red dotted square indicated slight discontinuity at the nozzle tip, graded as “slight scratch.” (c): red dotted square indicated obvious discontinuity at the nozzle tip, graded as “deep scratch.”

Figure 3 displays representative microscopic views of damage classification in IOL injectors in this study.

**Figure 4:**
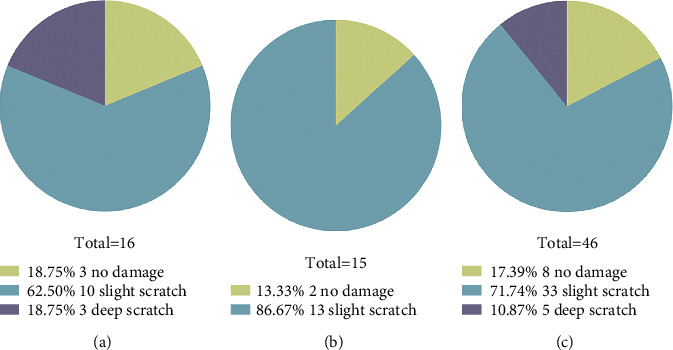
The distribution of damage profile of three injector models. (a) Accuject. (b) RayOne. (c) Bluemixs.

Figure 4 displays the distribution of damage profiles for three injector models. Accuject and Bluemixs showed similar damage distribution ranging from “no damage” to “deep scratch.” Although no statistically significant difference in terms of the damage scores was observed between the study groups, RayOne displayed a better damage distribution from “no damage” to “slight scratch.”

### 3.2. Parameters of Nozzle Tips and Plunger Tips

Table 1 summarizes the nozzle tip parameters for each injector model.

**Figure 5:**
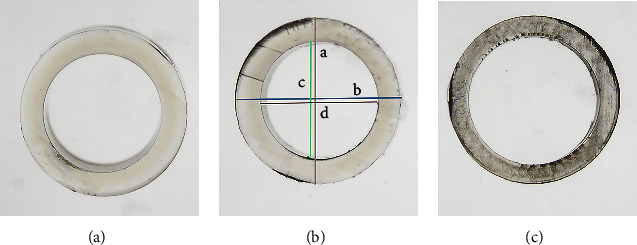
Representative microscopic images of cross-sectional surfaces for all injector models. *a* = outer cross-sectional vertical diameter, *b* = outer cross-sectional horizontal diameter, *c* = inner cross-sectional vertical diameter, *d* = inner cross-sectional horizontal diameter. (a) Accuject. (b) Bluemixs. (c) RayOne.

Table 2 summarizes the plunger tip parameters for each injector model. All three injector models showed similar outer cross-sectional diameters and similar outer cross-sectional areas. RayOne had a larger inner cross-sectional diameter and a larger inner cross-sectional area than the other groups.  Figure 5 displays exemplary microscopic views of the cross-sectional surfaces for each injector model.

**Figure 6:**
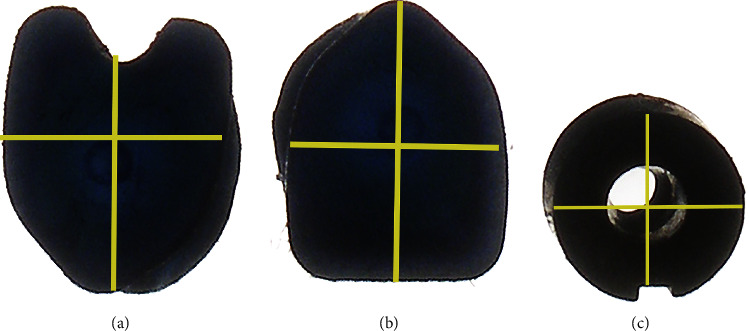
Representative microscopic images of plunger tips for all injector models. Yellow lines marked the vertical and horizontal diameters. (a) Bluemixs. (b) Accuject. (c) RayOne.

Figure 6 displays exemplary microscopic views of plunger tips for each injector model. RayOne had the smallest plunger tip parameter, while the other 2 groups had comparable parameters. The tip angles of the three injector models were 56° (Accuject), 56° (Bluemixs) and 44° (RayOne) (as shown in Figure 1).

## 4. Discussion

In this study, all IOLs were implanted with the injectors without any intraoperative complications. Under the operating microscope, the IOLs appeared to be undamaged, but the injectors showed various degrees of damage, from no damage to deep scratches. All three groups tend to fall into the category of minor to moderate damage. To our understanding, this is the first study to evaluate the tip damage of hydrophilic IOL injectors using a systemic damage scale.

When an IOL passes through the nozzle tip, the friction between the IOL and the nozzle tip, the IOL and the plunger, and the plunger and the nozzle tip can cause damage to the nozzle tip. In our previous study [12], we showed the diameters of the nozzle tip may have an impact on the extent of damage to the nozzle tip. Simply explained, the larger the internal cross-sectional area, the less friction there is when an IOL passes through the tip of the nozzle. Compared to the inner cross-sectional areas of the IOL models in our previous study [12, 13] (2.23 mm^2^ for Acrysert, 1.87 mm^2^ for Ultrasert and 1.81 mm^2^ for Autonome, 2.52 mm^2^ for iTec and Simplicity), the IOL models in this study had much smaller inner cross-sectional areas (1.16 mm^2^ for Bluemixs, 1.17 mm^2^ for Accuject and 1.38 mm^2^ for RayOne). However, the damage values for the IOL models in our study are similar to those of the preloaded injectors loaded with hydrophobic IOLs in the previous study. We speculate that this may be due to the difference between hydrophilic and hydrophobic acrylic IOLs. With higher water content, hydrophilic acrylic IOLs are more compressible and therefore cause less friction with the nozzle tips [14].

The injection force during IOL implantation undoubtedly played an important role in the extent of damage to the nozzle tip. In a previous study by Cabeza-Gil et al. [15] they concluded that for all injectors, plate hydrophilic IOLs have the lowest resistance forces; hydrated C-loop hydrophobic IOLs have higher forces; and the C-loop hydrophobic IOL under dry conditions has the highest resistance forces. This may explain why hydrophilic IOL injectors in this study caused minor to moderate damage to the nozzle tip after IOL implantation, even with relative smaller nozzle tip diameters compared to IOL injectors with hydrophobic IOLs [12, 13, 16]. Thus, we suspect that the greater the injection force, the greater the damage to the tip. We cannot confirm whether the nozzle tip materials differ between Bluemixs and Accuject. However, it appears that the nozzle tip configuration and parameters were the same for these two injector models. The identical configuration and parameters of these two injector models can also be employed to explain why the damage scores and distribution of damage categories were comparable for Bluemixs and Accuject.

Although there was no significant difference in the damage scores between the 3 groups, RayOne showed a better distribution of damage categories than the other 2 groups. This could be attributed to the larger inner cross-sectional diameters of RayOne. In a previous study [17], the authors concluded that the less acute the angle of the bevel tip, the less damage to the nozzle tip after IOL implantation. In this study, RayOne had a more acute tip angle than the other groups. However, RayOne did not cause more damage to the tip. We postulated that the cross-sectional diameters also play an important role in nozzle tip damage.

During the IOL implantation, the plunger is gently pushed or screwed forward to advance the IOL forward into the eyes. Therefore, plunger is critical to achieve a successful implantation. Currently, the plunger of an injector could be made of metal or plastic. According to an earlier study [6], the diameter of the plunger tips affects the interaction between the nozzle tip and IOL. If the tip is large, the IOL and nozzle tip are subjected to excessive forces, which may trap the trailing haptic. Small tips tend to bypass the optic part and cause the IOL to become trapped in the cartridge. The ratios between the areas of the plunger tips and inner cross-sectional areas of the nozzle tips represent the relative size of the plunger tip. RayOne had a smaller ratio, while the other 2 groups had similar ratios. This may explain why RayOne had a better distribution of damage categories, since there was smaller friction between the plunger tip and nozzle tip. Although the ratios were different among the three groups, we did not observe any adverse events during the implantation procedure, indicating that those plungers were neither too small nor too large. The plunger of the three groups is all plastic plungers. Compared with IOL injector models with metal plungers in our previous studies [12, 13], all groups in this study caused less damage score. We speculated that metal is stiffer than plastic and therefore more likely to scratch the inner walls. This is consistent with a previous study where hard plungers were more likely to cause nozzle tip damage than soft plungers [6].

Smaller incision sizes have always been a goal in cataract surgery. It is generally accepted that smaller incision sizes are associated with early rehabilitation, better intraocular pressure control, and low or negligible postoperative astigmatism and complications [18]. Incision size is largely related to the diameter of the nozzle tip [11]. In general, the smaller the diameter of the nozzle tip, the smaller the incision enlargement after IOL implantation. When a nozzle tip is fully inserted into a corneal incision, the corneal stretching is associated with the outer cross-sectional parameters [10]. Compared to the results from our other studies [12, 13], the cross-sectional areas in our study (RayOne: 2.28 mm^2^, Bluemixs: 2.24 mm^2^, Accuject: 2.24 mm^2^) are smaller than those from hydrophobic IOL injector models (Ultrasert: 2.57 mm^2^, Acrysert: 3.15 mm^2^, Monarch: 2.44 mm^2^, AutonoMe: 2.59 mm^2^, iTec: 2.51 mm^2^, Emerald cartridge: 3.46 mm^2^, Simplicity: 2.52 mm^2^). We suspect that this may be why the manufacturer claims that the incision size for Bluemixs can be as small as 1.8 mm [19], whereas the most commonly used incision sizes are typically 2.4–2.8 mm [20]. Therefore, the hydrophilic IOLs with smaller nozzle tip diameters should be considered when smaller incision sizes are desired.

The limitations of this research are listed as follows. First, this research employed IOLs in each group with various diopter. However, all the IOLs that were examined in our research had diopter ranges between +15D and +26D, which is the most common range to be used in clinical settings. In addition, there was no statistically significant difference in IOL dioptric power between the three groups. As a result, when the injectors are used in accordance with the manufacturer's directions, the impact of the different IOL diopters is negligible. Second, this study was retrospective in nature, and its primary goal was to present the various degrees of damage that can be done to hydrophilic IOL injectors after IOL implantation. There is still a need for more research to examine the relationship between the extent of injector damage and its clinical impact.

In summary, hydrophobic and hydrophilic IOLs are currently the most commonly used IOL models. It is important to understand the difference between the injector models of these two IOL categories. All three injector models for hydrophilic IOLs showed minor to moderate damage after IOL implantation, even with smaller diameter injector tips. RayOne showed a better distribution of damage categories. All three injector models had relatively small tip sizes, so if smaller incision sizes are required for certain patients, smaller tip parameters should be taken into consideration.

## Figures and Tables

**Table 1:** Nozzle tip parameters for each injector model.

Injector model	Outer cross-sectional vertical diameter (mm)	Outer cross-sectional horizontal diameter (mm)	Outer cross-sectional area (mm^2^)	Inner cross-sectional vertical diameter (mm)	Inner cross-sectional horizontal diameter (mm)	Inner cross-sectional area (mm^2^)
RayOne	1.70	1.71	2.28	1.33	1.32	1.38
Accuject	1.69	1.69	2.24	1.22	1.22	1.17
Bluemixs	1.69	1.69	2.24	1.21	1.22	1.16

**Table 2:** Plunger tip parameters for each injector model.

Injector model	Vertical diameter (mm)	Horizontal diameter (mm)	Area (mm^2^)	Plunger tip area/inner cross-sectional area of the nozzle tip (%)
RayOne	0.67	0.71	0.43	31.16
Accuject	1.05	0.83	0.78	66.67
Bluemixs	0.88	0.84	0.74	63.79

## Data Availability

The data used to support the findings in this study are available from the corresponding author upon reasonable request.

## Supplementary Materials

Supplementary Materials Supplemental Figure 1: Representative microscopic images of each damage scale. This is a direct copy from our previous study^12^ to demonstrate each damage scale. (a) No damage. (b) Red square indicates fine stress lines on the inner tube, graded as “slight scratches.” (c) Red square indicates slight discontinuity at the nozzle tip, graded as “slight scratches.” (d) Red square indicates deep stress lines on the inner tube, graded as “deep scratches.” (e) Red square indicates obvious discontinuity at the nozzle tip, graded as “deep scratches.” (f) Red square indicates partial crack of inner tube, graded as “extension.” (g) Red square indicates full thickness of inner tube crack, graded as “crack.” (h) Red square indicates burst of the nozzle tube, graded as “burst.” Supplemental Figure 2: IOL diopters in each study group (mean ± SD). No statistically significant difference was observed across three groups in terms of the dioptric powers of the IOLs (*P* > 0.05). Supplemental Table 1: Test IOLs used in this study.
