# Supplementary figures and images for: Evaluation of Parameters and Nozzle Tip Damage after Clinical Use of Three Hydrophilic Intraocular Lens Injector Models
Source: J Ophthalmol. 2024 May 30;2024:2360368. doi: 10.1155/2024/2360368 (PMC11156505; doi:10.1155/2024/2360368)

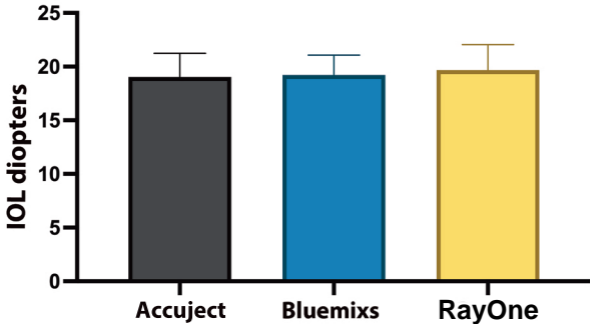

Supplement: Supplementary Materials — Supplemental Figure 1: Representative microscopic images of each damage scale. This is a direct copy from our previous study12 to demonstrate each damage scale. (a) No damage. (b) Red square indicates fine stress lines on the inner tube, graded as “slight scratches.” (c) Red square indicates slight discontinuity at the nozzle tip, graded as “slight scratches.” (d) Red square indicates deep stress lines on the inner tube, graded as “deep scratches.” (e) Red square indicates obvious discontinuity at the nozzle tip, graded as “deep scratches.” (f) Red square indicates partial crack of inner tube, graded as “extension.” (g) Red square indicates full thickness of inner tube crack, graded as “crack.” (h) Red square indicates burst of the nozzle tube, graded as “burst.” Supplemental Figure 2: IOL diopters in each study group (mean ± SD). No statistically significant difference was observed across three groups in terms of the dioptric powers of the IOLs (P > 0.05). Supplemental Table 1: Test IOLs used in this study. [file 2360368.f1.zip › supplemental figure 2 (1) (1).pdf]

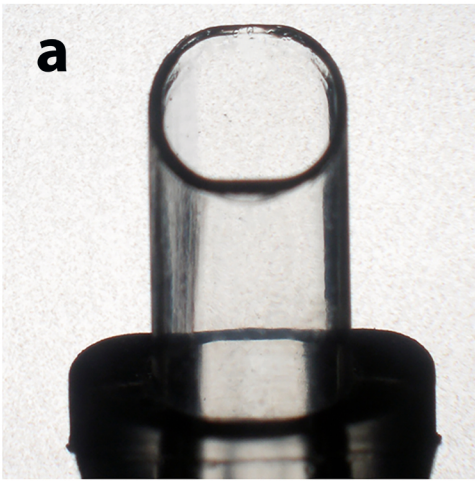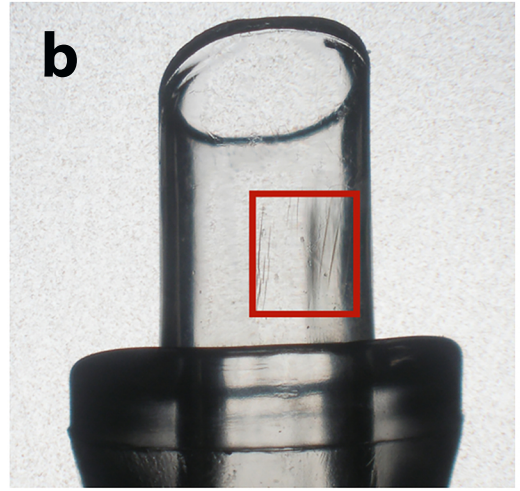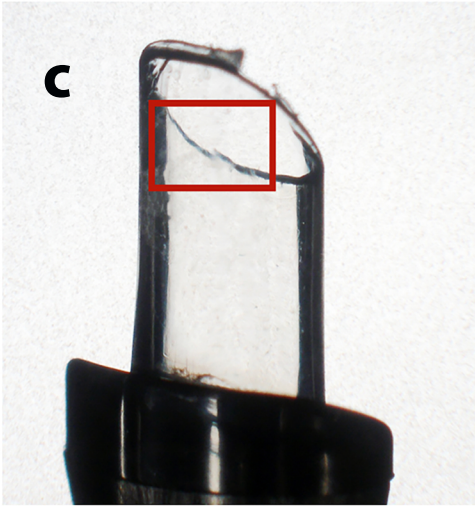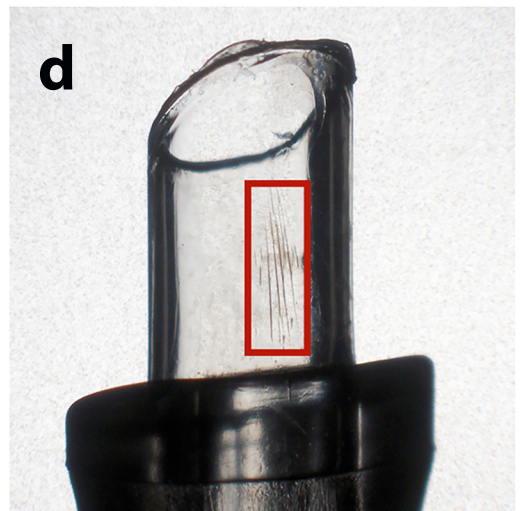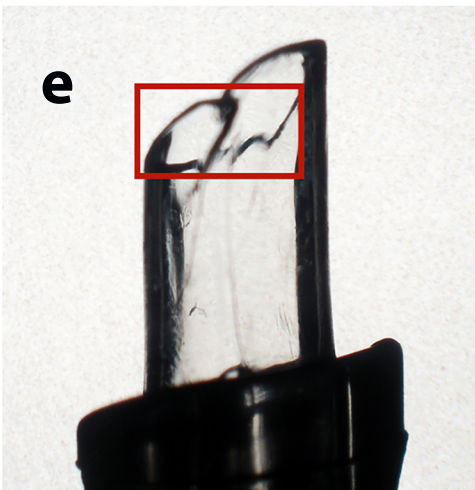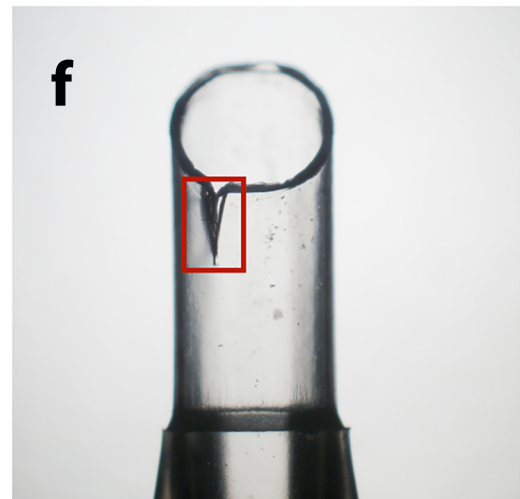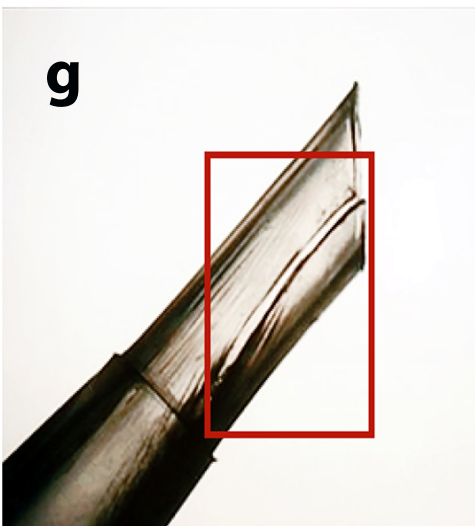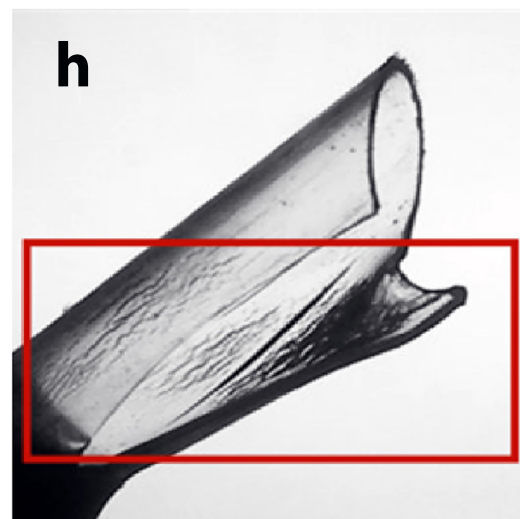

Supplement: Supplementary Materials — Supplemental Figure 1: Representative microscopic images of each damage scale. This is a direct copy from our previous study12 to demonstrate each damage scale. (a) No damage. (b) Red square indicates fine stress lines on the inner tube, graded as “slight scratches.” (c) Red square indicates slight discontinuity at the nozzle tip, graded as “slight scratches.” (d) Red square indicates deep stress lines on the inner tube, graded as “deep scratches.” (e) Red square indicates obvious discontinuity at the nozzle tip, graded as “deep scratches.” (f) Red square indicates partial crack of inner tube, graded as “extension.” (g) Red square indicates full thickness of inner tube crack, graded as “crack.” (h) Red square indicates burst of the nozzle tube, graded as “burst.” Supplemental Figure 2: IOL diopters in each study group (mean ± SD). No statistically significant difference was observed across three groups in terms of the dioptric powers of the IOLs (P > 0.05). Supplemental Table 1: Test IOLs used in this study. [file 2360368.f1.zip › supplmental figure 1 (1) (1).pdf]
